# Food Sources of Animal Protein in Relation to Overall and Cause-Specific Mortality—Causal Associations or Confounding? An Analysis of the EPIC-Heidelberg Cohort

**DOI:** 10.3390/nu15153322

**Published:** 2023-07-26

**Authors:** Rashmita Bajracharya, Rudolf Kaaks, Verena Katzke

**Affiliations:** Department of Cancer Epidemiology, German Cancer Research Center (DKFZ), 69120 Heidelberg, Germany; rashmita.bajracharya@dkfz.de (R.B.); r.kaaks@dkfz.de (R.K.)

**Keywords:** red meat, processed meat, poultry, dairy, mortality

## Abstract

While prior prospective iso-caloric substitution studies show a robust association between higher intake of animal protein and risk of mortality, associations observed for mortality risk in relation to major food sources of animal protein have been generally more diverse. We used the EPIC-Heidelberg cohort to examine if confounding, notably, by smoking, adiposity, or alcohol intake, could cause inconsistencies in estimated mortality hazard ratios (HR) related to intake levels of different types of meat and dairy products. Higher intakes of red or processed meats, and lower intakes of milk or cheese, were observed among current heavy smokers, participants with obesity, or heavy alcohol drinkers. Adjusting for age, sex, and total energy intake, risk models showed increased all-cause, cardiovascular, and cancer-related mortality with higher red or processed meat intakes (HR ranging from 1.25 [95% confidence interval = 1.15–1.36] to 1.76 [1.46–2.12] comparing highest to lowest tertiles), but reduced risks for poultry, milk, or cheese (HR ranging from 0.55 [0.43–0.72] to 0.88 [0.81–0.95]). Adjusting further for smoking history, adiposity indices, alcohol consumption, and physical activity levels, the statistical significance of all these observed was erased, except for the association of processed meat intake with cardiovascular mortality (HR = 1.36 [CI = 1.13–1.64]) and cheese intake with cancer mortality (HR = 0.86 [0.76–0.98]), which, however, were substantially attenuated. These findings suggest heavy confounding and provide little support for the hypothesis that animal protein, as a nutrient, is a major determinant of mortality risk.

## 1. Introduction

On an average population level, national statistics of food availability have documented strong correlations in average per capita availability and consumption levels of food types in relation to the economic development of different countries [1,2]. Related to these correlations in food consumption patterns, major shifts can also be observed in the average macronutrient composition of the diet [1,3]. One of these is a shift toward a higher percent of energy intake in the form of animal protein and fat, combined with reductions in energy intake from vegetable protein, as well as from (complex) carbohydrates [1,4]. Furthermore, international comparisons show strong, positive correlations between average per capita availability of animal protein sources and age-standardized incidence rates of chronic diseases, such as diabetes, heart disease, and stroke, and many forms of cancer (e.g., colon, lung, breast, and prostate) that are predominant in high-income countries [5]. These various observations led to the hypothesis that sub-optimal dietary composition of macronutrients—with a high intake of animal protein as one of its key characteristics—may be a contributing cause of chronic disease development and mortality. Paradoxically, however, economic development and sub-optimal dietary composition of macronutrients also show an association with reduced overall (all-cause) or cardiovascular mortality rates and better average life expectancy [6,7,8] but an increase in cancer mortality rates [9,10,11,12].

To examine whether the associations seen between animal protein and mortality risks at the ecological level are also observed on the level of single individuals, we [13] and several other research groups [14,15,16,17], have recently published findings from iso-caloric modeling analyses in the context of prospective cohort studies. These studies quite consistently indicated increased risks of all-cause and cardiovascular mortality, but not of cancer-related mortality, in association with higher proportions of dietary energy derived from animal protein [13,14,15,16,17], which seems to contradict observations from international correlation studies [5,6]. Further, potential contradictions also appear when examining mortality risk in association with consumption levels for individual food sources for animal protein. Here, studies have generally reported increased risks of all-cause, cardiovascular, and cancer-related mortality in relation to higher intakes of red and processed meat [18,19,20,21,22], whereas mostly inverse risk relationships have been found for consumption of poultry [20,23,24] or dairy products [25,26], with some degree of heterogeneity across different studies, particularly, for the association of dairy intake with all-cause, cardiovascular, and cancer mortality [27,28,29]. These heterogeneous findings across main food groups contributing to animal protein intake raise the question of whether animal protein itself, as a nutrient, is a genuine cause contributing to higher mortality risk. Furthermore, these findings raise the question of whether some of the observed associations of mortality risks with animal protein or its various food sources could have been mostly the result of confounding by other lifestyle-related risk factors, in particular, smoking, obesity, alcohol intake, or physical inactivity. To address this question in greater depth, and as a follow-up of our recent modeling of mortality endpoints in association with macro-nutrient intakes [13], we here present further findings from the EPIC-Heidelberg cohort, examining the global association patterns of diverse animal protein-rich foods with overall and cause-specific mortality outcomes, before and after adjustment for other major risk factors (smoking, adiposity, alcohol intake, education level, physical activity), to critically assess potential confounding patterns by the direction and magnitude.

## 2. Methods

### 2.1. Study Population: The EPIC-Heidelberg Cohort

EPIC-Heidelberg recruited participants and collected data between 1994 and 1998 as part of the larger European EPIC study [30,31]. The EPIC-Heidelberg cohort included 25,540 study participants aged 35–65 years recruited from the general population living in the southern German city of Heidelberg and its surrounding municipality [32]. Forty-six percent of the participants were men. After the exclusion of participants lost to follow-up after baseline ascertainment (*n* = 1171), those with prevalent cancer or myocardial infraction or diabetes diagnosis (*n* = 1159) at recruitment, those in extreme top and bottom 1 percentile of “energy intake/energy requirement” ratio (*n* = 442) calculated based on age, sex, weight, height, and physical activity level, and missing information on level of education (*n* = 20), 22,748 remained for the analysis. The data for those with unknown information about smoking history (*n* = 341) were imputed using a fully conditional specification multiple imputation method [33]. Baseline examinations included a detailed medical interview and comprehensive questionnaire assessments of environmental and behavioral factors and habitual diet. Anthropometric measurements were taken by trained personnel, and blood samples were obtained from 95% of the participants. All participants provided informed consent.

### 2.2. Assessment of Habitual Diet

Information about habitual diet was collected using a self-administered food frequency questionnaire (FFQ), which had been extensively validated in prior studies [33,34,35]. Briefly, a total of 158 single foods or mixed dishes were included. For each food item, the participant provided information about the consumption of the food in the past year (e.g., for the participants recruited in 1994, the past year would be 1993), frequency of consumption (1–6 times) and the time period (day, week, month or year). A semi-quantitative questionnaire was used, requesting information not only about the frequency of consumption but, for a number of food items, also about habitual portion sizes. To increase the accuracy of portion size estimation, photographs of food portions of various sizes were included. A food composition database [36] was used to convert food consumption data into estimated intakes of nutrients and total energy. The estimation of total energy and macronutrient intake based on the FFQ was also validated [33]. The definition of food groups for this analysis—namely, red meat, processed meat, poultry, cheese, and milk—is based on all suitable items from the FFQ. The list of items included within each food group is provided in Supplemental Table S1.

### 2.3. Prospective Ascertainment of Mortality Endpoints

The mortality outcomes were ascertained first through regular record linkages with municipal registries for vital status and then, for all cases of death, by collecting further information on causes of death (death certificates) from regional health offices. Causes of death, as derived from death certificates, were then coded according to ICD-10 by trained medical study personnel. When the relative risk reported in the literature of the association of smoking or alcohol intake with mortality outcome was greater than 2.5, it was regarded as strongly smoking-related mortality or strongly alcohol-related mortality [37,38,39,40,41]. The detailed ICD-10 codes used to create aggregated cancer outcomes are provided in Supplemental Table S2. The present analyses are based on complete case ascertainments from June 1994 to May 2019.

### 2.4. Statistical Analyses

Relative mortality hazards (hazard ratios (HR)) and 95% confidence intervals (CI) were estimated using Cox proportional hazard models and cause-specific Cox models, with age as the underlying time scale, to determine the association of lifestyle factors (including smoking status, waist circumference, BMI, level of education, physical activity, and alcohol consumption) and animal protein-rich foods with incident chronic disease and cause-specific mortality. For each disease endpoint of interest, age at exit was defined as age at diagnosis, age at last attendance to follow-up, death, or end of follow-up (May 2019), whichever came first. Food group scores were modeled as tertiles, with the lowest tertiles serving as reference categories. We computed risk estimates and 95% confidence intervals for a crude model adjusted only for age, sex, and total energy intake (as a continuous variable). To test for further potential confounding, model variations were generated that stepwise included additional covariates for smoking status (never, former [quit > 10 years], former [quit ≤ 10 years], current [≤15 cigarettes per day], current [>15 cigarettes per day], pipe/cigar/occasional), physical activity level (inactive, moderately inactive, moderately active, active), body mass index (kg/m^2^), waist circumference (cm), baseline alcohol intake (gram alcohol/day), and the level of formal education (university degree, secondary school, technical school, and primary school or none). We tested for linear trends of the associations by modeling the tertile categories as integer scores. To assess the magnitude of confounding by lifestyle variables, we calculated the percent change in the relative risk for disease or mortality endpoints—in the models that were stepwise adjusted for lifestyle covariates compared to the minimally adjusted model—in relation to food intake. To examine the association between animal protein-rich food groups and the selected lifestyle covariates, we examined the percent change in the context of mean difference in intake of each food group—estimated from generalized linear models, particularly MANOVA (multivariate analysis of variance) minimally adjusted for age, sex, and total dietary energy intake—by lifestyle variables. Statistical significance was defined as *p* < 0.05 or 95% confidence intervals excluding the null, and all analyses were performed using SAS Version 9.4 (SAS Institute, Inc., Cary, NC, USA).

## 3. Results

### 3.1. Cohort Characteristics

Among the 22,748 participants retained for the present analyses, a total of 3486 cases of deaths were registered until the end of the follow-up (May 2019), of whom 932 (26.7%) died of cardiovascular events, 1572 (45.0%) of cancer and the remaining 982 (28.1%) of other miscellaneous conditions (Table 1). Among cancer deaths, 365 (23.2%) died of strongly smoking-related cancers, and 73 (4.6%) died of strongly alcohol-related and smoking-related cancer. The median age of the participants at recruitment was 51.1 (Inter-Quartile Range [IQR] = 43.5–57.5) years, and 53% were female. Almost 43% of the participants never smoked, and 44% of the participants had a BMI < 25. 

### 3.2. Association of Non-Dietary Lifestyle Factors with Mortality

The results in Table 2 showed that smoking—when comparing current heavy smokers to never smokers—increased the risk of overall mortality (HR = 3.62 [95% CI = 3.29–3.98]) as well as cause-specific mortality; the risk was particularly high for strongly smoking-related cancer mortality (HR = 20.77 [14.76–29.22]). Next to smoking, alcohol intake was also associated with mortality due to alcohol-related (upper aero-digestive tract) cancers (currently moderately high versus currently low, HR = 16.73 [6.05–46.25]), as well as mortality due to the broader category of smoking-related tumors (HR = 4.13 [2.67–6.39]). Further to smoking and alcohol intake, measures of excess body weight (waist circumference and BMI) showed the strongest associations with cardiovascular mortality (waist circumference, HR = 2.29 [1.95–2.69]; BMI, HR = 2.44 [2.04–2.91]). Higher level of physical activity and formal education both consistently predicted lower risk of overall and cause-specific mortality. 

### 3.3. Association of Lifestyle Factors with Animal Protein-Rich Food-Groups

Table 3 provides an overview of the intake of animal protein-rich food groups across lifestyle risk factors, adjusted for age, sex, and total energy intake. Up to 64% higher intake of red meat and up to 49% higher intake of processed meat was observed among those in the highest adiposity category compared to the lowest. Higher intake of red or processed meat (from 22% up to 66%) was also seen among current heavy smokers, among participants with only primary school or lower education level, or among heavy alcohol drinkers compared to never smokers, participants with university degree, or never alcohol drinkers, respectively. In contrast, intake of cheese or milk was much lower for heavy alcohol drinkers (−30.2%) and for low formal education (−22.1% to −24%) and higher adiposity categories (−3.5% to −11.2%) compared to never drinkers, those with a university degree, and lower adiposity categories, respectively. Poultry intake was higher among those in higher adiposity categories (from 22% to 31%) and heavy alcohol drinkers (up to 23%) compared to least obese and never alcohol drinkers, respectively.

### 3.4. Association of Animal Protein-Rich Foods with Mortality

In basic risk models adjusting only for age, sex, and total energy intake (Table 4, Model 1), higher intake of red or processed meat was associated with an increase in the risk of all-cause mortality, overall cancer mortality, strongly alcohol and smoking-related cancer mortality, other cancer-related mortality (except smoking or alcohol-related cancer mortality) and cardiovascular mortality (HR ranging from 1.25 [1.15–1.36] to 1.76 [1.46–2.12]). In contrast, higher intake of poultry, milk, or cheese was associated with a lower risk of all-cause mortality, overall cancer mortality, strongly smoking-related cancer mortality, other cancer-related mortality, and cardiovascular mortality (HR ranging from 0.55 [0.43–0.72] to 0.88 [0.81–0.95]). After stepwise adjustment for smoking, alcohol consumption, physical activity, BMI, waist circumference, and formal education, most associations became statistically insignificant (Table 4, Model 2). The only associations that stayed significant after maximal adjustment were the higher risk of cardiovascular mortality in relation to processed meat intake (third compared to first tertile, HR = 1.36 [1.13–1.64]) and the lower risk of total cancer mortality in relation to consumption of cheese (second compared to first tertile, HR = 0.86 [0.76–0.98]) or milk (third compared to first tertile, HR = 0.83 [0.73–0.93]), although these associations were also substantially attenuated (by 15%) after confounder adjustments, compared to models minimally adjusted for age, sex, and total energy intake (Table 5).


**10–20%**

**20–30%**

**30%**
BMI and waist circumference adjusted


Smoking adjusted


Smoking, BMI, waist circumference and education adjusted


Fully adjusted




## 4. Discussion

Prior iso-caloric substitution studies in prospective study cohorts, including the EPIC-Heidelberg cohort, have consistently shown associations between higher intake of animal protein and increased overall and cardiovascular mortality, but not cancer mortality [13,14,16,42]. However, our present findings on the level of food groups do not unequivocally support the hypothesis that animal protein could be a major common risk factor for overall cardiovascular or cancer-specific mortality. In models minimally adjusted for age, sex, and total energy intake, contrasting associations were observed for overall and cause-specific mortality risks with intake levels of different food groups contributing to animal protein—red and processed meats, poultry, and dairy products. Specifically, red or processed meat increased the risk of overall as well as cardiovascular and cancer-specific mortality, whereas poultry or dairy products reduced the risk. Furthermore, and importantly, most of the associations were attenuated to the point of no longer being statistically significant when models were adjusted further for smoking history, adiposity, alcohol consumption, and physical activity.

It is worth noting that the associations of food intake levels with overall and cause-specific mortality found in the EPIC-Heidelberg cohort, contrasting as they were for different main sources of animal protein, are mostly in line with findings from other prospective studies. Although differences in the time period, study population, type of dietary assessment tools used, or covariate adjustments may have led to heterogeneity across prospective studies worldwide, meta-analyses showed mostly higher risks of overall cardiovascular and cancer-related mortality in relation to red meat or processed meat intake and lower risks in relation to consumption levels of poultry, cheese or milk [19,20,21,23,24,25,26,43,44,45], even though findings for dairy products have been more diverse across different studies [27,28,29]. We found that most of the associations disappeared after considering mortality endpoints grouped by their known relationships with smoking, alcohol intake, and adiposity and when careful adjustments were made for the latter risk factors. The only two associations remaining after these adjustments were an increased risk of cardiovascular mortality in relation to processed meat and a lower risk for overall cancer-related mortality in association with the intake of dairy products, although these remaining associations were attenuated after the adjustments. It is also worth noting that in past meta-analyses of prospective studies, similar to our findings, processed meat intake consistently showed a strong association with all-cause mortality, particularly cardiovascular mortality [21,45], while the association with unprocessed red meat was not systematically observed [46]. 

With regard to overall confounding patterns for specific food groups, we found substantially higher intakes of red meat, processed meat, and poultry among current heavy smokers and among those with higher levels of obesity or alcohol intake, which are all major risk factors for cardiovascular disease or cancer. By contrast, intake levels of milk were considerably lower among regular alcohol consumers. Additionally, we also observed that higher consumption levels of red and processed meats, and lower consumption of cheese and milk, were characteristic of individuals with lower levels of formal education. Overall, we speculate that, in part, the above association patterns could be explained by differences in income as a further characteristic of social stratification; unfortunately, however, we have no data on household income in the EPIC-Heidelberg cohort and, therefore, could not analyze this aspect further.

One limitation of this study is that our primary exposure, i.e., intakes of foods rich in animal protein, was assessed at only one point in time, at the baseline recruitment. Therefore, there is a chance for the participants to have changed their dietary habits or lifestyle, which could result in attenuated associations. In terms of strength, this was a large prospective cohort study with detailed information collected about such lifestyle habits as smoking, alcohol consumption, and BMI, which are strong confounders for the association of food intake with disease and mortality. Thus, we were able to adjust for all important known confounders of the association. That said, we do not exclude the possibility that some residual confounding by these factors remained due to inaccuracies in their measurements. 

In summary, we found no convincing evidence that any of the principal food sources of animal protein are meaningful determinants for overall cardiovascular or cancer-related mortality risk, independently of smoking, alcohol consumption, and excess body weight. Crucially, adjusting for the latter risk factors in the statistical models completely eliminated most associations of food consumption levels with mortality risk, leaving only two weak associations, of which one suggested a small increase in cardiovascular mortality (association with processed meat intake), and one suggested a weak reduction in cancer mortality (association with cheese consumption). Our present findings call into question whether the cumulative intake of animal protein from various food sources combined is a plausible risk determinant, in contrast to suggestive findings obtained via the iso-caloric substitution modeling [14,15,16,17,42].

## Figures and Tables

**Table 1:** Baseline characteristics of sampled EPIC-Heidelberg participants (*n* = 22,748).

Characteristics	Total *n* (%)	Men *n* (%)	Women *n* (%)
*n*	22,748	10,600 (46.6)	12,148 (53.4)
Age at recruitment (years, inter-quartile range)	51.1 (43.5–57.5)	52.8 (46.4–58.1)	48.7 (41.7–56.7)
Smoking intensity			
Never	9722 (42.7)	3545 (33.4)	6177 (50.8)
Former (quit > 10 years)	5208 (22.8)	3005 (28.3)	2203 (18.1)
Former (quit ≤ 10 years)	2509 (11.0)	1329 (12.5)	1180 (9.7)
Current (≤15 cig. Per day)	2550 (11.2)	921 (8.6)	1629 (13.4)
Current (>15 cig. Per day)	2339 (10.2)	1402 (13.2)	937 (7.7)
Pipe/cigar/occasional	420 (1.8)	398 (3.7)	22 (0.1)
Waist circumference level ^a^			
Low waist circumference	11,016 (48.4)	4673 (44.0)	6343 (52.2)
Moderate waist circumference	5922 (26.0)	3204 (30.2)	2718 (22.3)
High waist circumference	5810 (25.5)	2723 (25.6)	3087 (25.4)
Body mass index			
<25	10,040 (44.1)	3297 (31.1)	6743 (55.5)
≥25–<30	9120 (40.0)	5491 (51.8)	3629 (29.8)
≥30	3588 (15.7)	1812 (17.0)	1776 (14.6)
Level of formal education			
University degree	6962 (30.6)	3952 (37.2)	3010 (24.7)
Secondary school	1639 (7.2)	594 (5.6)	1045 (8.6)
Technical school	7709 (33.8)	2826 (26.6)	4883 (40.2)
Primary school or none	6438 (28.3)	3228 (30.4)	3210 (26.4)
Physical activity level			
Inactive	2590 (11.3)	1129 (10.6)	1461 (12.0)
Moderately inactive	7951 (34.9)	3575 (33.7)	4376 (36.0)
Moderately active	6563 (28.8)	3076 (29.0)	3487 (28.7)
Active	5644 (24.8)	2820 (26.6)	2824 (23.2)
Alcohol consumption			
Never	342 (1.5)	74 (0.7)	268 (2.2)
Former	851 (3.7)	436 (4.1)	415 (3.4)
>0–6 (M)/>0–3 (W)	5384 (23.6)	1282 (12.0)	4102 (33.7)
>6–12 (M)/>3–12 (W)	6769 (29.7)	1614 (15.2)	5155 (42.4)
>12–24	4680 (20.5)	3042 (28.7)	1638 (13.4)
>24	4722 (20.7)	4152 (39.1)	570 (4.6)
Total energy intake (kcal), mean, SD	1971.3 (632.0)	2223.5 (666.1)	1751.3 (506.9)
Red meat (g/d), mean, SD	31.7 (29.7)	41.6 (35.0)	23.0 (20.6)
Processed meat (g/d), mean, SD	51.8 (40.6)	64.4 (45.8)	40.9 (31.6)
Poultry (g/d), mean, SD	12.5 (14.1)	13.9 (15.4)	11.3 (12.7)
Cheese (g/d), mean, SD	29.8 (21.6)	29.4 (22.6)	30.1 (20.6)
Milk (g/d), mean, SD	82.4 (138.8)	81.9 (154.1)	82.9 (124.0)
Overall death	3486 (15.3)	2259 (21.3)	1227 (10.1)
Cardiovascular death	932 (4.1)	649 (6.1)	283 (2.3)
Cancer death	1572 (6.9)	972 (9.1)	600 (4.9)
Strongly smoking-related cancer deaths	365 (1.6)	263 (2.4)	102 (0.8)
Strongly alcohol-related cancer deaths	73 (0.3)	58 (0.5)	15 (0.1)
Other deaths	982 (4.3)	638 (6.0)	344 (2.8)

^a^ Low waist circumference = <80 cm in women and <94 cm in men; moderate waist circumference = 80–<88 cm in women and 94–<102 cm in men; high waist circumference = ≥88 cm in women and ≥102 cm in men.

**Table 2:** HRs (95% CI) for the relationships of co-variables with risks of mortalities (*n* = 22,748).

		Overall Mortality *n*_CASES_ = 3486 HR (95% CI) ^a^	Cardiovascular Mortality *n*_CASES_ = 932 HR (95% CI)	Cancer Mortality				Other Mortality *n*_CASES_ = 982 HR (95% CI)
				Overall Cancer Mortality *n*_CASES_ = 1572 HR (95% CI)	Strongly Smoking-Related Cancer Deaths *n*_CASES_ = 365 HR (95% CI)	Strongly Smoking and Alcohol-Related Cancer Deaths *n*_CASES_ = 73 HR (95% CI)	Other Cancer-Related Mortality ^b^ *n*cases = 1207 HR (95% CI)	
Smoking intensity	Never	Ref	Ref	Ref	Ref	Ref	Ref	Ref
Model 1	Former (quit > 10 years)	1.07 (0.98–1.17)	1.03 (0.86–1.23)	1.11 (0.97–1.28)	1.97 (1.32–2.93) *	1.68 (0.79–3.56)	1.04 (0.89–1.21)	1.01 (0.85–1.21)
	Former (quit ≤ 10 years)	1.46 (1.30–1.64) *	1.41 (1.11–1.78) *	1.42 (1.19–1.70) *	3.75 (2.41–5.82) *	2.41 (0.99–5.87)	1.21 (0.99–1.49)	1.39 (1.10–1.75) *
	Current (≤15 cig. Per day)	2.07 (1.85–2.30) *	2.12 (1.70–2.64) *	1.86 (1.57–2.21) *	6.46 (4.32–9.65) *	2.35 (0.89–6.23)	1.47 (1.21–1.79) *	2.18 (1.77–2.69) *
	Current (>15 cig. Per day)	3.62 (3.29–3.98) *	3.65 (3.02–4.42) *	3.52 (3.04–4.07) *	20.77 (14.76–29.22) *	10.41 (5.26–20.59) *	1.97 (1.63–2.37) *	3.76 (3.12–4.53) *
Waist circumference level ^c^	<80/<94	Ref	Ref	Ref	Ref	Ref	Ref	Ref
Model 1	80–<88/94<102	1.16 (1.07–1.26) *	1.42 (1.19–1.70) *	1.11 (0.98–1.26)	0.87 (0.67–1.12) *	0.57 (0.31–1.08)	1.23 (1.06–1.42) *	1.01 (0.85–1.20)
	≥88/≥102	1.73 (1.61–1.87) *	2.29 (1.95–2.69) *	1.42 (1.26–1.60) *	1.13 (0.88–1.44)	1.08 (0.63–1.85)	1.55 (1.35–1.78) *	1.82 (1.57–2.11) *
Body mass index level	<25	Ref	Ref	Ref	Ref	Ref	Ref	Ref
Model 1	≥25–<30	1.13 (1.05–1.23) *	1.34 (1.14–1.59) *	1.12 (0.99–1.25)	0.82 (0.65–1.03)	0.63 (0.38–1.06)	1.27 (1.11–1.46) *	1.02 (0.87–1.19)
	≥30	1.76 (1.62–1.93) *	2.44 (2.04–2.91) *	1.45 (1.26–1.67) *	0.80 (0.59–1.10)	0.78 (0.40–1.54)	1.75 (1.49–2.05) *	1.75 (1.48–2.07) *
Level of formal education	University degree	Ref	Ref	Ref	Ref	Ref	Ref	Ref
Model 1	Secondary school	1.48 (1.26–1.73) *	1.55 (1.11–2.17) *	1.41 (1.11–1.78) *	1.42 (0.79–2.56)	1.91 (0.62–5.89)	1.38 (1.07–1.79) *	1.48 (1.08–2.02) *
	Technical school	1.41 (1.29–1.55) *	1.43 (1.18–1.74) *	1.36 (1.18–1.56) *	2.26 (1.66–3.09) *	2.03 (1.02–4.07) *	1.21 (1.04–1.41) *	1.51 (1.26–1.80) *
	Primary school or none	1.81 (1.66–1.97) *	2.18 (1.83–2.61) *	1.60 (1.40–1.83) *	3.03 (2.25–4.09) *	3.15 (1.65–6.01) *	1.39 (1.20–1.62) *	1.76 (1.48–2.09) *
								
Model 2	Secondary school	1.32 (1.13–1.54) *	1.37 (0.98–1.92)	1.27 (1.01–1.61) *	1.10 (0.61–1.98)	1.59 (0.51–4.92)	1.29 (1.00–1.67) *	1.32 (0.97–1.81)
	Technical school	1.22 (1.12–1.34) *	1.19 (0.98–1.45)	1.21 (1.05–1.39) *	1.85 (1.35–2.53) *	1.88 (0.93–3.78)	1.10 (0.94–1.29)	1.30 (1.08–1.55) *
	Primary school or none	1.50 (1.37–1.64) *	1.72 (1.43–2.06) *	1.39 (1.21–1.59) *	2.46 (1.81–3.35) *	2.92 (1.50–5.69) *	1.22 (1.05–1.43) *	1.44 (1.21–1.72) *
Physical activity level	Inactive	Ref	Ref	Ref	Ref	Ref	Ref	Ref
Model 1	Moderately inactive	0.68 (0.62–0.74) *	0.75 (0.62–0.90) *	0.78 (0.67–0.92) *	0.66 (0.48–0.90) *	0.70 (0.33–1.49)	0.82 (0.69–0.98) *	0.53 (0.44–0.63) *
	Moderately active	0.65 (0.58–0.71) *	0.62 (0.51–0.76) *	0.82 (0.70–0.96) *	0.71 (0.51–0.98) *	0.85 (0.40–1.82)	0.86 (0.72–1.03)	0.50 (0.41–0.60) *
	Active	0.68 (0.61–0.75) *	0.65 (0.52–0.79) *	0.77 (0.65–0.91) *	0.65 (0.46–0.91) *	0.84 (0.39–1.83)	0.82 (0.68–0.99) *	0.61(0.51–0.74) *
Alcohol consumption ^d^	Currently low	Ref	Ref	Ref	Ref	Ref	Ref	Ref
Model 1	Currently moderately low	1.46 (1.14–1.87) *	1.49 (0.90–2.45)	1.01 (0.64–1.59)	1.01 (0.31–3.26)	-	1.09 (0.67–1.79)	1.83 (1.18–2.86) *
	Currently moderately high	2.29 (1.99–2.64) *	2.00 (1.49–2.69) *	2.02 (1.61–2.54) *	4.13 (2.67–6.39) *	16.73 (6.05–46.25) *	1.64 (1.25–2.16) *	2.89 (2.24–3.74) *
	Currently high	0.86 (0.77–0.95) *	0.80 (0.65–0.99) *	0.90 (0.77–1.05)	1.04 (0.72–1.50)	1.67 (0.58–4.83)	0.86 (0.72–1.02)	0.85 (0.69–1.04)
	Former	0.90 (0.81–1.01)	0.87 (0.69–1.08)	0.96 (0.81–1.14)	0.98 (0.66–1.45)	1.53 (0.51–4.52)	0.96 (0.80–1.16)	0.89 (0.72–1.11)
	Never	1.40 (1.26–1.56) *	1.39 (1.13–1.72) *	1.42 (1.21–1.67) *	2.16 (1.51–3.07) *	3.61 (1.34–9.76) *	1.24 (1.03–1.50) *	1.42 (1.16–1.74) *

Note: * *p*-value < 0.05; Model 1 was adjusted for age at recruitment (in continuous years) and sex. Model 2 was, in addition to model 1, adjusted for smoking status, waist circumference, body mass index, physical activity, and alcohol intake. ^a^ Hazard ratios (HR) and 95% confidence intervals (CI) are based on Cox proportional hazards models (for overall mortality) and cause-specific Cox models (for cardiovascular mortality, cancer mortality, and other mortality). ^b^ Other cancer-related mortality refers to all cancer-related deaths except smoking or alcohol-related cancer mortality. ^c^ Low waist circumference = <80 cm in women and <94 cm in men; moderate waist circumference = 80–<88 cm in women and 94–<102 cm in men; high waist circumference = ≥88 cm in women and ≥102 cm in men. ^d^ Alcohol consumption in g alcohol/day (currently low = >0–3 in women and >0–6 in men; currently moderately low = >3–12 in women and >6–12 in men; currently moderately high = >12–24; currently high = >24).

**Table 3:** Change in mean energy-adjusted intakes of foods by categories of co-variables ^a^.

		Red Meat (Grams/Day)		Processed Meat (Grams/Day)		Poultry (Grams/Day)		Cheese (Grams/Day)		Milk (Grams/Day)	
		Mean (% Difference)		Mean (% Difference)		Mean (% Difference)		Mean (% Difference)		Mean (% Difference)	
Smoking status	Never	29.4		48.6		12.3		29.6		80.8	
	Former (quit > 10 years)	+2.4	+8.1%	+3.3	+6.8%	+0.2	+1.6%	+1.1	+3.7%	−3.4	−4.2%
	Former (quit ≤ 10 years)	+2.3	+7.8%	+5.1	+10.5%	+1	+8.1%	+1.3	+4.3%	+1.9	+2.3%
	Current (≤15 cig. Per day)	+0.7	+2.3%	+3.2	+6.6%	−0.2	−1.6	+0.6	+2.0%	+4.9	+6.0%
	Current (>15 cig. Per day)	+13	+44.2%	+14.7	+30.3%	+0.7	+5.6%	−3.1	−10.4%	+15.6	+19.3%
Waist circumference ^b^	<80/<94	27.2		45.6		11.5		31		86.2	
	80–<88/94–<102	+6.6	+24.4%	+9.1	+19.9%	+1.3	+11.3%	−2.3	−7.4%	−9.7	−11.2%
	≥88/≥102	+11.7	+43.3%	+15.3	+33.5%	+2.6	+22.6%	−2.4	−7.7%	−5.0	−5.8%
Body mass index	<25	25.3		43.2		11.1		31.6		85.4	
	25–<30	+9.9	+39.2%	+12.9	+29.7%	+2.1	+18.9%	−3.2	−10.1%	−6.3	−7.3%
	≥30	+16.2	+64.2%	+21.5	+49.6%	+3.5	+31.5%	−3.4	−10.7%	−3.0	−3.5%
Educational level	University degree	29.9		46.8		12.4		34		94.6	
	Secondary school	−2.7	−9.0%	−0.5	−1.0%	+0.5	+4.0%	−0.5	−1.4%	+2.5	+2.6%
	Technical school	+0.3	+1.0%	+4.3	+9.1%	0	0%	−5.8	−17.0%	−17.6	−18.6%
	Primary school or no formal education	+6.7	+22.7%	+12.9	+27.5%	+0.6	+4.8%	−8.2	−24.0%	−21.6	−22.1%
Physical activity	Active	30.4		51.8		12.2		30.4		91.6	
	Moderately active	+1	+3.2%	−0.4	−0.7%	+0.4	+3.2%	−0.2	−0.6%	−12.6	−13.7%
	Moderately inactive	+1.7	+5.5%	+0.4	+0.7%	+0.4	+3.2%	−0.9	−2.9%	−13	−14.1%
	inactive	+3.6	+11.8%	+0.2	+0.3%	+0.4	+3.2%	−1.9	−6.2%	−8.9	−9.7%
Alcohol intake ^c^	Never	26.6		44.6		11.1		26.8		107.2	
	Former	+4.8	+17.9%	+9.5	+20.8%	+1.3	+11.2%	+2.2	+8.2%	−6.2	−5.9%
	>0–6 (M)/>0–3 (W)	−1.2	−4.4%	−1.7	−3.7%	−0.3	−2.5%	+2.0	+7.4%	−15.6	−14.8%
	>6–12 (M)/>3–12 (W)	−0.4	−1.4%	−0.8	−1.7%	+0.3	+2.5%	+3.6	+13.4%	−22.1	−21.0%
	>12–24	+7.6	+28.4%	+9.8	+21.5%	+1.2	+10.3%	+3.5	+13.0%	−25.9	−24.7%
	>24	+17.7	+66.2%	+22.3	+49.0%	+2.7	+23.2%	+3.0	+11.1%	−31.7	−30.2%

^a^ Adjusted for age, sex, and total energy intake. ^b^ Low waist circumference = <80 cm in women and <94 cm in men. Moderate waist circumference = 80–<88 cm in women and 94–<102 cm in men. High waist circumference = ≥88 cm in women and ≥ 102 cm in men. ^c^ Currently low alcohol intake = <5 g alcohol/day; currently moderately low alcohol intake = 5–<15 g alcohol/day; currently moderately high alcohol intake = 15–<30 g alcohol/day); currently high alcohol intake = ≥30 g alcohol/day; 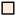 +10–<+20%, 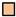 +20–<+30%, 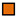 +30–<+40%, 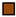 +40–<+50% (adverse association of food group with co-variable); 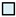 −10–<−20%, 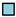 −20%-<−30%, 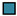 −30–<−40% (advantageous association of food group with co-variable.

**Table 4:** HRs (95% CI) as well as *p_trend_* values for associations between food groups and mortalities (*n* = 22,748).

		Overall Mortality *n*_CASES_ = 3768	Cardiovascular Mortality *n*_CASES_ = 932	Cancer Mortality				Other Mortality *n*_CASES_ = 982
				Overall Cancer Mortality *n*_CASES_ = 1572	Strongly Smoking-Related Cancer Deaths *n*_CASES_ = 365	Strongly Smoking and Alcohol-Related Cancer Deaths *n*_CASES_ = 73	Other Cancer-Related Mortality ^a^ *n*cases = 1207	
Red meat								
Model 1 ^b^	1st tertile	Ref	Ref	Ref	Ref	Ref	Ref	Ref
	2nd tertile	1.02 (0.94–1.11)	1.18 (0.98–1.41)	1.00 (0.88–1.14)	0.94 (0.71–1.26)	0.86 (0.45–1.66)	1.03 (0.88–1.19)	0.92 (0.78–1.09)
	3rd tertile	1.25 (1.15–1.36) *	1.40 (1.17–1.67) *	1.20 (1.05–1.37)	1.20 (0.91–1.58)	1.04 (0.56–1.93)	1.21 (1.04–1.40) *	1.20 (1.01–1.41) *
	*p_trend_*	<0.001	<0.001	0.004	0.13	0.80	0.01	0.01
Model 2 ^c^	2nd tertile	0.92 (0.85–1.01)	1.02 (0.85–1.22)	0.93 (0.82–1.07)	0.86 (0.64–1.15)	0.82 (0.42–1.59)	0.96 (0.82–1.11)	0.86 (0.72–1.02)
	3rd tertile	1.00 (0.92–1.09)	1.04 (0.86–1.24)	1.00 (0.88–1.15)	0.90 (0.68–1.20)	0.88 (0.46–1.66)	1.03 (0.88–1.21)	0.98 (0.83–1.17)
	*p_trend_*	0.72	0.66	0.81	0.58	0.75	0.59	0.90
Processed meat								
Model 1	1st tertile	Ref	Ref	Ref	Ref	Ref	Ref	Ref
	2nd tertile	1.09 (1.00–1.19) *	1.31 (1.09–1.57) *	1.12 (0.98–1.27)	1.23 (0.92–1.63)	1.41 (0.67–2.96)	1.09 (0.94–1.26)	0.99 (0.84–1.16)
	3rd tertile	1.27 (1.17–1.39) *	1.76 (1.46–2.12) *	1.20 (1.05–1.38) *	1.29 (0.96–1.73)	2.14 (1.05–4.37) *	1.20 (1.03–1.40) *	1.11 (0.94–1.32)
	*p_trend_*	<0.001	<0.001	0.007	0.09	0.025	0.01	0.19
Model 2	2nd tertile	0.98 (0.90–1.07)	1.13 (0.94–1.36)	1.04 (0.91–1.19)	1.09 (0.81–1.45)	1.30 (0.61–2.77)	1.02 (0.88–1.18)	0.89 (0.75–1.05)
	3rd tertile	1.06 (0.97–1.16)	1.36 (1.13–1.64) *	1.06 (0.92–1.22)	1.09 (0.81–1.48)	1.04 (0.98–4.26)	1.06 (0.90–1.24)	0.92 (0.77–1.09)
	*p_trend_*	0.16	<0.001	0.41	0.57	0.037	0.46	0.39
Poultry								
Model 1	1st tertile	Ref	Ref	Ref	Ref	Ref	Ref	Ref
	2nd tertile	0.97 (0.90–1.05)	1.01 (0.86–1.18)	0.97 (0.86–1.09)	0.71 (0.55–0.92) *	1.03 (0.60–1.77)	1.07 (0.93–1.22)	0.93 (0.80–1.08)
	3rd tertile	0.93 (0.86–1.00)	0.89 (0.76–1.05)	0.93 (0.83–1.06)	0.84 (0.66–1.08)	0.63 (0.34–1.15)	0.97 (0.84–1.12)	0.88 (0.75–1.03)
	*p_trend_*	0.07	0.20	0.31	0.18	0.13	0.73	0.11
Model 2	2nd tertile	0.99 (0.91–1.07)	1.02 (0.87–1.19)	0.99 (0.87–1.11)	0.80 (0.62–1.04)	1.15 (0.66–1.98)	1.07 (0.93–1.22)	0.95 (0.81–1.10)
	3rd tertile	0.92 (0.85–1.00)	0.87 (0.74–1.02)	0.95 (0.84–1.07)	0.98 (0.76–1.26)	0.75 (0.41–1.37)	0.95 (0.82–1.09)	0.88 (0.75–1.03)
	*p_trend_*	0.06	0.09	0.43	0.88	0.36	0.48	0.11
Cheese								
Model 1	1st tertile	Ref	Ref	Ref	Ref	Ref	Ref	Ref
	2nd tertile	0.85 (0.79–0.92) *	0.80 (0.68–0.93) *	0.79 (0.70–0.89) *	0.64 (0.50–0.82) *	0.73 (0.41–1.29)	0.85 (0.74–0.98) *	1.06 (0.91–1.23)
	3rd tertile	0.80 (0.74–0.87) *	0.80 (0.68–0.94) *	0.79 (0.70–0.89) *	0.55 (0.43–0.72) *	0.69 (0.40–1.22)	0.87 (0.76–1.00)	0.81 (0.69–0.96) *
	*p_trend_*	<0.001	0.005	<0.001	<0.001	0.20	0.05	0.01
Model 2	2nd tertile	0.94 (0.87–1.02)	0.90 (0.76–1.05)	0.86 (0.76–0.98) *	0.80 (0.62–1.03)	0.90 (0.50–1.59)	0.91 (0.79–1.04)	1.16 (1.00–1.35)
	3rd tertile	0.94 (0.87–1.02)	0.96 (0.82–1.13)	0.91 (0.80–1.03)	0.78 (0.60–1.02)	0.98 (0.55–1.74)	0.95 (0.83–1.10)	0.96 (0.82–1.14)
	*p_trend_*	0.14	0.61	0.13	0.06	0.93	0.51	0.79
Milk								
Model 1	1st tertile	Ref	Ref	Ref	Ref	Ref	Ref	Ref
	2nd tertile	0.87 (0.81–0.94) *	0.84 (0.71–0.98) *	0.89 (0.79–1.00)	0.82 (0.63–1.05)	0.86 (0.50–1.46)	0.90 (0.78–1.03)	0.88 (0.75–1.02)
	3rd tertile	0.88 (0.81–0.95) *	0.91 (0.78–1.07)	0.83 (0.73–0.93) *	0.81 (0.63–1.04)	0.57 (0.32–1.03)	0.82 (0.71–0.94)	0.86 (0.74–1.00)
	*p_trend_*	0.001	0.23	0.002	0.09	0.068	0.006	0.06
Model 2	2nd tertile	0.92 (0.85–0.99)	0.87 (0.75–1.03)	0.94 (0.83–1.06)	0.91 (0.71–1.17)	0.94 (0.55–1.62)	0.93 (0.81–1.06)	0.93 (0.80–1.08)
	3rd tertile	0.95 (0.88–1.03)	0.99 (0.85–1.16)	0.89 (0.79–1.01)	0.93 (0.72–1.19)	0.67 (0.37–1.21)	0.87 (0.75–1.00)	0.93 (0.80–1.09)
	*p_trend_*	0.23	0.86	0.08	0.55	0.19	0.05	0.40

Note: The exposure variables were divided into cohort-wide tertiles, with the first tertile serving as the reference category. * *p*-value < 0.05, *p_trend_* < 0.05. ^a^ Other cancer-related mortality refers to all cancer-related deaths except smoking or alcohol-related cancer mortality. ^b^ Model 1 adjusted for age, sex, and total energy intake. ^c^ Model 2 stratified by age, sex, total energy intake, smoking, physical activity, alcohol intake, BMI, waist circumference, and formal education.

**Table 5:** Magnitude of confounding.

		Overall Mortality *n*_CASES_ = 3768	Cardiovascular Mortality *n*_CASES_ = 932	Cancer Mortality				Other Mortality *n*_CASES_ = 982
				Cancer Mortality *n*_CASES_ = 1572	Strongly Smoking-Related Cancer Deaths *n*_CASES_ = 365	Strongly Smoking and Alcohol-Related Cancer Deaths *n*_CASES_ = 73	Other Cancer-Related Mortality ^a^ *n*_CASES_ = 1207	
Red meat	BMI and waist circumference	10.4%	16.4%	5.8%	5.8%	15.3%	9.0%	11.6%
	Smoking	8%	8.5%	8.3%	22.5%	18.2%	4.1%	8.3%
	Smoking, BMI, waist circumference, and education	19.2%	25.7%	15.8%	24.1%	15.3%	14.0%	20%
	Fully adjusted	20%	25.7%	16.6%	25%	15.3%	14.8%	18.3%
Processed meat	BMI and waist circumference	10.2%	15.9%	5.8%	6.2%	14.0%	9.1%	11.7%
	Smoking	3.9%	3.9%	4.1%	12.4%	9.3%	1.6%	4.5%
	Smoking, BMI, waist circumference, and education	17.3%	22.7%	12.5%	18.6%	10.7%	12.5%	18.9%
	Fully adjusted	16.5%	22.7%	11.6%	15.5%	51.4%	11.6%	17.1%
Poultry	BMI and waist circumference	5.3%	6.7%	2.1%	3.5%	6.3%	4.1%	4.5%
	Smoking	3.2%	4.4%	4.3%	10.7%	9.5%	2.0%	3.4%
	Smoking, BMI, waist circumference, and education	2.1%	3.3%	1.0%	15.4%	15.8%	3.0%	1.1%
	Fully adjusted	1.0%	2.2%	2.1%	16.6%	19.0%	2.0%	0%
Cheese	BMI and waist circumference	2.5%	3.7%	1.2%	1.81%	4.3%	2.2%	3.7%
	Smoking	7.5%	8.7%	7.5%	25.4%	20.2%	3.4%	8.6%
	Smoking, BMI, waist circumference, and education	16.2%	20%	13.9%	40%	36.2%	8.0%	17.2%
	Fully adjusted	17.5%	20%	15.1%	78.1%	42.0%	9.1%	18.5%
Milk	BMI and waist circumference	2.2%	3.2%	1.2%	1.23%	1.7%	1.2%	2.3%
	Smoking	1.1%	2.1%	1.2%	4.9%	5.2%	1.2%	2.3%
	Smoking, BMI, waist circumference, and education	5.6%	7.6%	4.8%	9.8%	10.5%	3.6%	5.8%
	Fully adjusted	7.9%	8.7%	7.2%	14.8%	17.5%	6.0%	8.1%

Percentage change in relative risk for third tertile vs. the first tertile after adjustment compared to minimally adjusted (for age, sex, and total energy intake) model. ^a^ Other cancer-related mortality refer to all cancer-related deaths except smoking or alcohol-related cancer mortality.

## Data Availability

Access to the data used for the analysis will be made available at request. Please contact the corresponding author.

## Supplementary Materials

The following supporting information can be downloaded at: https://www.mdpi.com/article/10.3390/nu15153322/s1, Table S1: Food items included in each food group; Table S2: Types of cancer categories; Table S3: Coefficient of determination (R^2^) ^a^ showing the proportion of variance in food sources of animal protein intake that can be explained by lifestyle variables.
