# Supplementary material for: Food Sources of Animal Protein in Relation to Overall and Cause-Specific Mortality—Causal Associations or Confounding? An Analysis of the EPIC-Heidelberg Cohort
Source: Nutrients. 2023 Jul 26;15(15):3322. doi: 10.3390/nu15153322 (PMC10421322; doi:10.3390/nu15153322)
Supplement: Supplementary file 1 [file nutrients-15-03322-s001.zip › nutrients-2500156-supplementary.pdf]

Supplementary Table S1: Food items included in each food group

Food Group	Food Items
Red meat	Beef, veal, pork, mutton/lamb, horse, goat
Processed meat	Bacon, liver containing items, ham, hamburger, meatballs, minced meat, other processed meat (example: sausage, canned meat)
Poultry	Chicken, turkey, duck, goose, rabbit
Cheese	Ricotta, other cheeses (example: cheddar, gouda, camembert, mozzarella)
Milk	Whole milk, semi-skimmed milk, skimmed milk, non-specified milk, fermented milk

Supplementary Table S2: Types of cancer categories

	Cancer Sites	ICD-10 Codes
Strongly smoking-related cancer deaths	Oral cavity and pharynx	C001, C01, C021, C023, C030, C031, C040, C048, C049, C051, C069, C07, C080, C089, C098, C099, C102, C103, C108, C109, C12, C132, C138, C148
	esophagus	C150, C153, C154, C155, C158, C159, C14, C15
	larynx	C320, C321, C322, C328, C329
	Trachea, lung, bronchus	C33, C340, C3402, C341, C342, C343, C348, C349
	nose, nasal cavity, and middle ear	C300, C301, C310, C311, C319
	mediastinum and other respiratory organs	C383
Strongly alcohol- and smoking related cancer deaths	Oral cavity and pharynx	C001, C01, C021, C023, C030, C031, C040, C048, C049, C051, C069, C07, C080, C089, C098, C099, C102, C103, C108, C109, C12, C132, C138, C148
	esophagus	C150, C153, C154, C155, C158, C159, C14, C15
	larynx	C320, C321, C322, C328, C329

Supplementary Table S3: Coefficient of determination (R^2) [a] showing the proportion of variance in food sources of animal protein intake that can be explained by lifestyle variables.

	Smoking Status (R^2)	Measures of Adiposity [b] (R^2)	Alcohol Intake (R^2)	Physical Activity (R^2)	Smoking Status, MEASURES of adiposity, Alcohol Intake, and Physical Activity (R^2)	Smoking Status, Measures of ADIPOSITY, alcohol Intake, Physical Activity, and Education (R^2)
Red meat **(g/day)**	0.17	0.19	0.17	0.18	0.21	0.21
Processed meat **(g/day)**	0.25	0.28	0.25	0.25	0.28	0.29
Poultry **(g/day)**	0.03	0.04	0.03	0.03	0.04	0.04
Cheese **(g/day)**	0.07	0.08	0.07	0.07	0.08	0.10
Milk **(g/day)**	0.03	0.03	0.04	0.03	0.04	0.04

[a] R^2 values were estimated using linear regression models adjusted for age, sex and total energy intake

[b] Measures of adiposity include measures of waist circumference and body mass index
